# MicroRNA miR-509-3p inhibit metastasis and epithelial-mesenchymal transition in hepatocellular carcinoma

**DOI:** 10.1080/21655979.2021.1932210

**Published:** 2021-06-11

**Authors:** Huiming Zhang, Shuang Liu, Liqiang Chen, Yanliang Sheng, Wenzhe Luo, Gang Zhao

**Affiliations:** a School of Basic Medicine, Jiamusi University, Jiamusi, China; b School of Stomatology, Jiamusi University, Jiamusi, China

**Keywords:** Hepatocellular carcinoma (HCC), miR-509-3p, Twist, epithelial–mesenchymal transition (EMT), invasion, metastasis

## Abstract

Our study seeks to obtain data which help to assess the impacts and related mechanisms of microRNA miR-509-3p in hepatocellular carcinoma (HCC). We found that the expression of miR-509-3p was down-regulated and Twist was up-regulated in HCC tissues and cell lines (HepG2, HCCLM3, Bel7402, and SMMC7721) compared with the adjacent normal tissues and normal human hepatocyte (L02). Moreover, cell proliferation, invasion, migration and epithelial–mesenchymal transition (EMT) in HepG2 and HCCLM3 cells were appeared to be markedly suppressed by overexpressed miR-509-3p. Overexpression of miR-509-3p also performed inhibition of the growth and metastasis in vivo. In addition, miR-509-3p could target and inhibit Twist expression, and it could further reverse the tumor promotion by Twist in HCC. All in all, miR-509-3p overexpression causes inhibition of the proliferation, migration, invasion and EMT of HCC cells by negatively regulating Twist, thereby suppressing HCC development and metastasis.

## Graphical abstract

**Figure:**
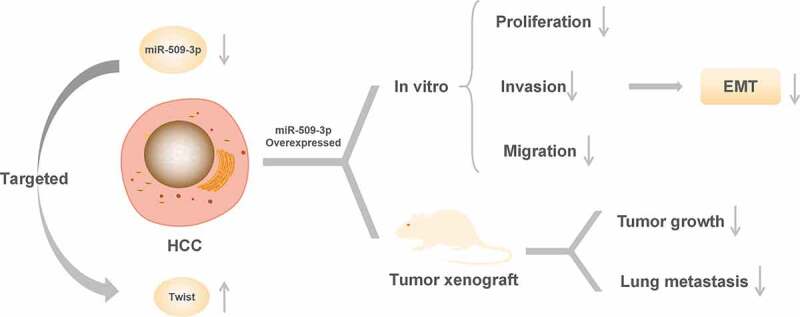

## Introduction

Liver cancer, a major contributor of cancer-related death [1], is described as the fifth most fatal cancer worldwide [2]. This disease can be classified as primary or secondary. Primary liver cancer originates from the epithelial or mesenchymal tissue of the liver. Secondary liver cancer, on the other hand, is mainly formed when other tumors metastasize to the liver, which is less common [3]. Hepatocellular carcinoma (HCC) has a high proportion among primary liver cancer, nearly accounting for about 70%-90% [4]. In China, approximately 50% of the total number of cases and deaths per year is contributed by HCC [5]. Over the past 10 years, surgical and pharmacologic treatment of liver cancer has improved much, such as the targeted therapy using kinase inhibitors (like sorafenib or regorafenib) for advanced HCC [6]. Despite these improvements, uncontrolled proliferation and metastasis of cancer cells result in rapid development of the cancer [7], and side effects and tumor drug resistance limit the clinical application of drugs [8]. Therefore, liver cancer patients still have low long-term survival rate [9]. For HCC, lung metastasis is one of the primary reasons why its survival is poor [10]. Hence, the mechanisms involved in HCC development are in urgent need of exploration to identify new molecules for targeted therapy and to improve clinical efficacy.

As non-coding RNAs, microRNAs (miRNAs) bind the 3ʹ-UTR of mRNAs for protein-coding gene regulation and signal transmission [11]. Their importance in tumor biology has been widely recognized [12]. And because of their specific molecular pathological features, articles have reported the successful application of miRNA expression profiles in the classification of different tumors or different stages of tumor [13]. In previous studies, both inhibition of TGF-β receptor and fibroblast growth factor expression by miR-140-5p and down-regulation of fibroblast growth factor 5 expression by miR-188-5p for suppressing HCC growth and metastasis were found [14,15]. In addition, it was also proved that in HCC cells, promotion of epithelial–mesenchymal transition (EMT) by miR-331-3p led to promotion of proliferation and metastasis of the cells [16]. Collectively, miRNAs are pivotal to HCC development.

MiR-509-3p has been reported to be an antitumor gene in a variety of cancers, inhibiting proliferation, migration, and promoting cisplatin-induced apoptosis [17,18]. For example, in ovarian cancer, the up-regulation of miR-509-3p in multicellular spheroids attenuated cellular proliferation and migration [19]. In osteosarcoma, the up-regulation of miR-509-3p caused inhibition of cellular migration, invasion, as well as proliferation, and enhancement of the sensitivity of osteosarcoma cells to cisplatin (CDDP) [20]. Notably, Li et al. reported that Long noncoding RNA 467 (LINC00467) promotes axitinib resistance in HCC by regulating miR-509-3p/PDGFRA axis [21]. It is evident that miR-509-3p is able to influence tumor development by affecting the biological activity of tumor cells. However, the effect of miR-509-3p on HCC metastasis has not been specifically reported.

Therefore, we hypothesized that the oncogene miR-509-3p could inhibit the growth and metastasis of HCC. And we carried out trials in vitro and in vivo for investigating specific effects and mechanism of miR-509-3p in order for a novel molecule and a new strategy for using in the clinical treatment of HCC.

## Materials and methods

### Clinical specimen collection

HCC (HCC group) and paracancerous tissues (Normal group) were collected from 60 HCC patients diagnosed and treated in our hospital. The patients were further split into lymph node metastasis group (LNM group) and non-metastasis group (non-LNM group). With informed consent from each patient, this study was carried out after obtaining the approval from the ethics committee of our hospital.

### Cell culture and transfection

Normal human hepatocyte (L02) and HCC cell lines (HepG2, HCCLM3, Bel7402, and SMMC7721) were purchased from American Type Culture Collection (ATCC, USA). All cell lines were cultured in the RPMI-1640 (Gibco, UAS) containing 10% fetal bovine serum (FBS, Gibco), 1% penicillin/streptomycin (Gibco), and maintained in a 5% CO_2_ incubator (Thermo, USA) at 37°C.

After that, following the steps of the lipo 3000 (Invitrogen, USA), HepG2 and HCCLM3 cells were transfected with negative mimics (NC mimics, 5ʹ-CUGCGUACGAGUACGCGU-3ʹ), miR-509-3p mimics, negative pcDNA3.1 (NC), and pcDNA3.1-Twist. The transfected cells were grouped into NC mimics, miR-509-3p, NC mimics+NC, miR-509-3p+NC, NC mimics+Twist, and miR-509-3p+Twist groups.

### qRT-PCR

Total RNA was extracted from HCC tissues and cells using the TRizol (Invitrogen, USA), followed by NanoDrop-based detection of the concentration and purity of RNA. The random primer reverse transcription kit (Thermo) cDNA was applied to prepare cDNA. The instructions of SYBR GREEN kit (Japan, TaKaRa) were followed to determine miR-509-3p and Twist mRNA expressions. The U6 and GAPDH were used as internal reference. Relative quantification of target gene expression by 2^−ΔΔCt^ was based on the obtained data. Table 1 presents the primer sequences.

**Table 1.** Primer sequences

RNA	Sequences (5 ‘to 3ʹ)
miR-509-3p	F: 5 ‘- TCGGCAGGUACUGCAGACGUG
	R: 5 ‘- CACTCAACTGGTGTCGTGGA
Twist	F: 5 ‘- CAGCAAGATCCAGACGCTCAAG
	R: 5 ‘- ACACGGAGAAGGCGTAGCTGAG
U6	F: 5 ‘- CTCGCTTCGGCAGCACA
	R: 5 ‘- AACGCTTCACGAATTTGCGT
GAPDH	F: 5 ‘- TCCCATCACCATCTTCCA
	R: 5 ‘- CATCACGCCACAGTTTTCC

### MTT assay

In 96-well plates, 1000 treated cells in logarithmic growth phase were plated into per well, followed by 24, 48, and 72 h culture. After that, another 4 h incubation was carried out with 20 μL of 5 mg/mL MTT solution in each well. Subsequently, the supernatant was aspirated, followed by a supplement of 150 μL of DMSO and 15 min shaking. Optical density (570 nm) was finally measured by using a microplate reader.

### Colony formation assay

Cell digestion using 0.25% trypsin was followed by cell resuspension using RPMI 1640 complete medium containing 0.35% agarose. Subsequently, in 6-well plates containing 0.6% agarose, 1 × 10^5^ cells were plated in per well, followed by cell culture in a 5% CO_2_ incubator at 37°C. When colonies were observed macroscopically, the culture was terminated. Finally, after staining step using 0.1% crystal violet solution, the photographing step was completed under an inverted microscope for counting the number of colonies.

### Transwell assay

The Matrigel-coated upper chamber was placed in a 5% CO_2_ incubator at 37°C for 30 min. Subsequently, the addition of 100 μL of cells in the upper chamber, while the lower chamber with 700 μL of medium containing 20% FBS, followed by the application of a 5% CO_2_ incubator for cell culture at 37℃ for 12–24 h. After the incubation, transwell inserts were rinsed three times using PBS. Then, 30-min fixation with 1% glutaraldehyde was carried out, followed by wiping off Matrigel and uninvaded cells and staining using 0.1% crystal violet. Finally, after being rising with PBS and drying, 6–10 fields were randomly observed under an upright microscope for counting and analyzing the number of positive cells within each field.

### Wound healing assay

After cell transfection completed in six-well plates, scratches perpendicular to the horizontal line were made with a sterile 10 μL tip. Cell debris and non-attached cells were removed by washing with PBS. Another 24-hour incubation with an addition of a fresh serum-free medium was carried out. Finally, photographs were taken with an inverted microscope, and Image J software was employed to calculate scratched areas.

### Dual-luciferase reporter assay

After the predication of the binding site of miR-509-3p to Twist by the bioinformatics website, wild-type (WT) and mutant (MUT) sequences of the binding site were inserted into the downstream of the firefly luciferase gene to construct the expression vector (USA, Promega). Recombinant plasmids consisting of miR-509-3p mimics and pmirGLO-Twist-WT/MUT and recombinant plasmids consisting of mimics NC and Twist-WT/MUT were transfected into 293 T cells using Lipo2000. On the completion of the 48-hour transfection, the final step is the assessment of luciferase activity utilizing dual-luciferase reporter assay kit.

### Western blot assay

Cell lysis buffer for extraction of total cellular protein was utilized, and then a BCA kit was applied for quantification of the extracted protein. Then, on completion of denaturing step by boiling in the sample loading buffer (1×), separation of 20 μg protein was conducted using SDS-PAGE. The protein, which subsequently blotted on PVDF membrane, was followed by a 1-hour blocking step using 5% skim milk powder. After that, co-incubation of the membrane and primary antibodies was performed overnight at 4°C followed by a rinsing step for three times and another 1-hour incubation with secondary antibodies at room temperature. Prior to adding chemiluminescence reagents to develop the protein, the membrane was washed again. The final step was to obtain the images using a gel imaging system and to analyze gray values of the protein bands using Image J software. Relative quantification was allowed with GAPDH as an internal reference.

### Tumorigenesis in nude mice

There were two groups of three BALB/c male nude mice each. The age of the mice was 4 to 6 weeks. HepG2 cells with different treatments were subcutaneously injected into the axilla. The mice with injection were named as NC mimics group and miR-509-3p group, respectively. Since subcutaneous injection, measurements of the length and width of the tumor using a vernier caliper were carried out every 4 days, which allowing the calculation of tumor volume (tumor volume = 0.5 × length × width^2^). Twenty-four days later, after complete anesthesia by intraperitoneal injection of 10% chloral hydrate, the mice were sacrificed by cervical dislocation. What to do next was the assessment of the final weight of the removed tumors. This trial was carried out on obtaining approval from the ethics committee and conformed to animal ethics.

### Lung metastasis test

There were two groups of three BALB/c male nude mice each. The age of the mice was 4 to 6 weeks. HepG2 cells with different treatments were intravenously injected into the tail vein. The mice with injection were named as NC mimics group and miR-509-3p group, respectively. Thirty days later, after complete anesthesia by intraperitoneal injection of 10% chloral hydrate, the mice were sacrificed by cervical dislocation. What to do next was the assessment of the final weight of the removed lung tissues. This trial was carried out on obtaining approval from the ethics committee and conformed to animal ethics.

### HE staining

After perfusion and fixation of the mouse lung tissue by using 4% paraformaldehyde, the left lobe of lung tissues was placed in 4% paraformaldehyde for fixation for 12 h. Subsequently, tissue samples were embedded and sectioned with a thickness of 15–25 mm, followed by a three-time rinsing with PBS. On completion of 5 min hematoxylin staining as well as following differentiation with hydrochloric acid alcohol, ammonia (1% volume fraction) was used for blueing the sections. Then, after rinsing, the sections carried out 30 s eosin staining and dehydration with alcohol. The final step was to clear the sections with xylene and mount. Observation of the tumor nodules in the lung tissues was performed under a biomicroscope. Based on three fields photographed, the number of nodules was statistically analyzed.

### Statistics

By using SPSS 26.0, one-way analysis of variance and independent samples T-test analysis were performed. The outcomes were all expressed in the form of mean ± standard deviation (SD). Pearson correlation was conducted for correlation analysis. Significant differences were suggested if P < 0.05.

## Results

### Lowly expressed miR-509-3p in hepatocellular carcinoma

**Figure 1.**
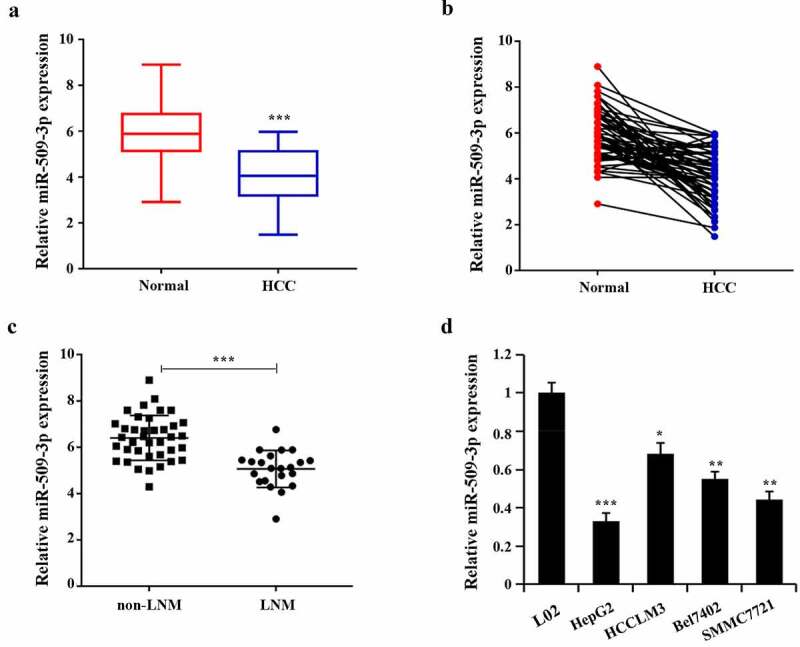
Lowly expressed miR-509-3p in hepatocellular carcinoma

Marked reduction of miR-509-3p expression in HCC tissues was found (Figure 1(a,b)). By comparison with the non-LNM group, miR-509-3p expression in the LNM group reduced (Figure 1(c)). Additionally, the confirmation of significantly decreased miR-509-3p expression was also achieved in HCC cells (HepG2, HCCLM3, Bel7402, SMMC7721) compared with the normal human hepatocyte (L02), with the relatively lowest expression in HepG2 and the relatively highest in HCCLM3 (Figure 1(d)). Together, these results indicate the possibility of the participation of miR-509-3p in HCC progression.

(a,b) Determination of miR-509-3p in paracancerous and hepatocellular carcinoma tissues using qRT-PCR; ***P < 0.001. (c) Evaluation of miR-509-3p in the tissues of the lymph node metastasis group (LNM) and non-lymph node metastasis group (non-LNM) using qRT-PCR; ***P < 0.001. (d) Detection of miR-509-3p in hepatocellular carcinoma cell lines using qRT-PCR; *P < 0.05, **P < 0.01, ***P < 0.001 vs. NHC group.

### Inhibition of hepatocellular carcinoma cell proliferation by miR-509-3p

**Figure 2.**
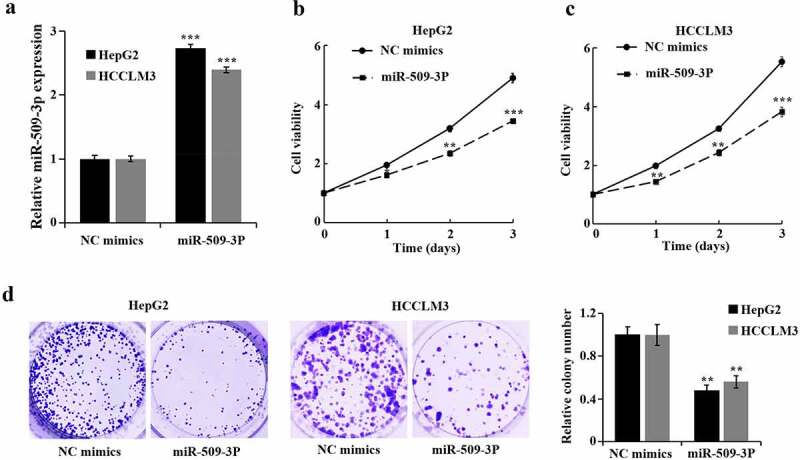
Inhibition of hepatocellular carcinoma cell proliferation by miR-509-3p

Transfection of miR-509-3p mimics and NC mimics into HepG2 and HCCLM3 cells was carried out for the purpose of determination of miR-509-3p functions in HCC cells. After 48 hours, miR-509-3p group showed a significant increase of miR-509-3p (Figure 2(a)). Additional assays revealed a marked overexpressed miR-509-3p-caused reduction in HCC cell proliferation and viability (Figure 2(b,c)).

(a) Determination of miR-509-3p expression in HepG2 and HCCLM3 cells using qRT-PCR. (b) Evaluation of cell proliferation by MTT assay. (c) Assessment of cell viability by colony formation assay. *P < 0.05, **P < 0.01 and ***P < 0.001 vs. NC mimics group.

### Inhibition of metastasis and epithelial–mesenchymal transition (EMT) in hepatocellular carcinoma cells by miR-509-3p

**Figure 3.**
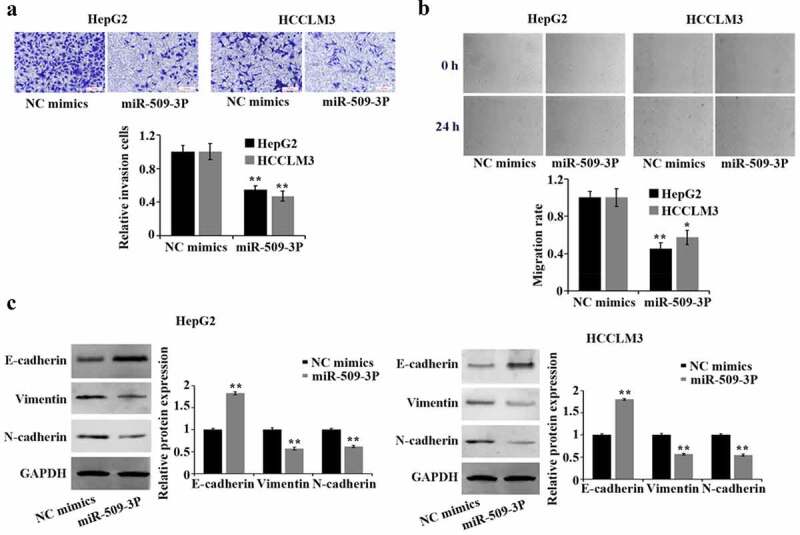
Inhibition of metastasis and epithelial–mesenchymal transition (EMT) in hepatocellular carcinoma cells by miR-509-3p

By comparison with NC mimics group, marked reduction of HCC cell invasion and migration was found in the miR-509-3p group (Figure 3(a,b)). EMT is pivotal in malignant transformation of cancer cells. Western blot-based results confirmed a significant increase of E-cadherin and a reduction of vimentin and N-cadherin expression in HepG2 and HCCLM3 cells after overexpression of miR-509-3p (Figure 3(c)). The involvement of miR-509-3p in HCC cells in suppressing metastasis and EMT was proved by the above acquired findings.

(a). Transwell assay for the determination of cell invasion using Transwell assay. (b) Utilization of wound healing assay for assessment of cell migration. (c) Western blot for evaluation of epithelial–mesenchymal transition (EMT) related proteins. *P < 0.05, **P < 0.01 and ***P < 0.001 vs. NC mimics group.

### Twist is a direct target gene of miR-509-3p

**Figure 4.**
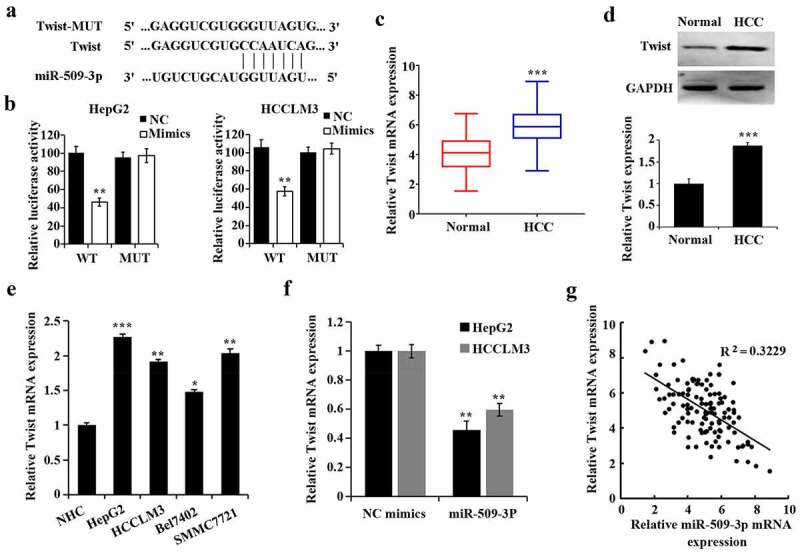
MiR-509-3p targets Twist

According to the prediction by TargetScan 7.2 software (http://www.targetscan.org/vert_72/), Twist could be one of the target genes of miR-509-3p (Figure 4(a)). Dual-luciferase reporter assay was utilized to verify the predication. Decreased luciferase activity of the Twist WT reporter vector caused by miR-509-3p mimics was found and thus confirmed that miR-509-3p targeted Twist (Figure 4(b)). Additionally, both increases of mRNA and protein of Twist in HCC tissues (Figure 4(c,d)) and Twist expression in HCC cell lines (Figure 4(e)) was confirmed.

By comparison with the NC mimics group, overexpressed miR-509-3p caused a reduction of Twist (Figure 4(f)). By utilizing Pearson correlation analysis, MiR-509-3p was further found to be of a negative correlation with Twist in HCC (Figure 4(g)). These findings suggested that miR-509-3p targeted Twist in HCC.

(a) The putative binding site of miR-509-3p and Twist (Position 370–376 of Twist 3ʹUTR) was shown. (b) Confirmation of miR-509-3p targeting Twist by dual-luciferase reporter assay; **P < 0.01 vs. NC group. (c) Determination of miR-509-3p expression in paracancerous and hepatocellular carcinoma (HCC) tissues utilizing qRT-PCR; ***P < 0.01 vs. Normal group. (d) Evaluation of Twist protein expression in paracancerous and HCC tissues using qRT-PCR; ***P < 0.001 vs. NC mimics group. (e) Determination of Twist expression in HCC cells using qRT-PCR; *P < 0.05, **P < 0.01 and ***P < 0.001 vs. NHC group. F. qRT-PCR-based evaluation of Twist expression in HCC cells after overexpression of miR-509-3p; **P < 0.01 vs. NC mimics group. G. MiR-509-3p was of a negative relationship with Twist in HCC cells.

### MiR-509-3p targets Twist to inhibit hepatocellular carcinoma progression

**Figure 5.**
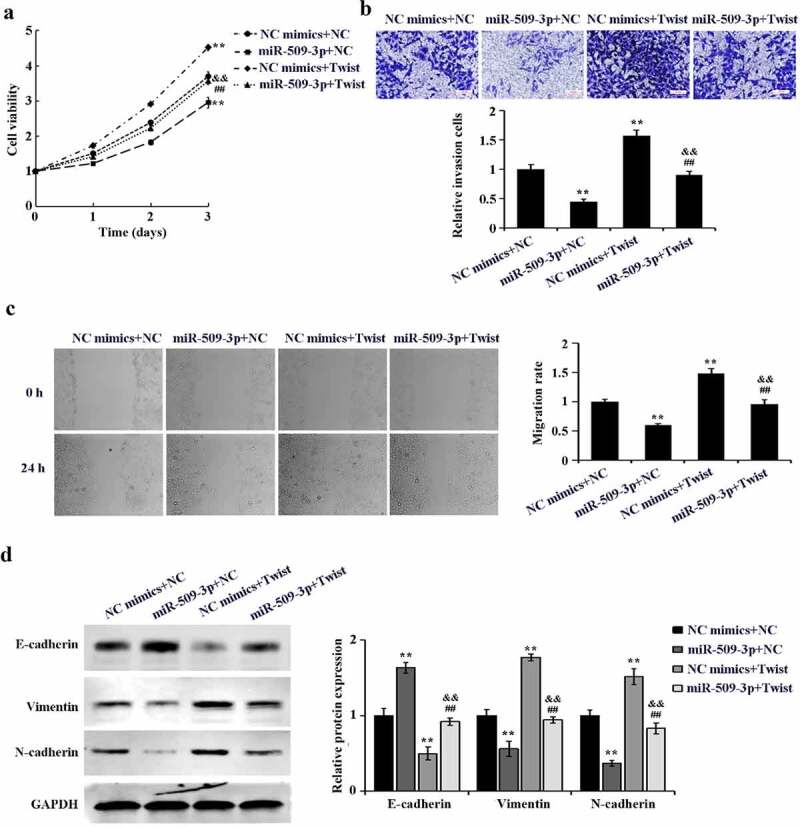
MiR-509-3p reverses the promotion of Twist on hepatocellular carcinoma progression

Cell rescue experiment was further performed to probe that miR-509-3p targets Twist to function in HCC. MiR-509-3p mimics and pcDNA3.1-Twist were co-transfected in HepG2 cells. By comparison with the NC mimics+NC group, marked increases of cell proliferation, invasion, migration and EMT were detected in the NC mimics+Twist group. Additionally, cell proliferation, invasion, and migration, as well as EMT in the miR-509-3p+Twist group presented a significant up-regulation by comparison with the miR-509-3p+NC group. However, the above biological behaviors showed marked down-regulation in the miR-509-3p+Twist group as compared to the NC mimics+Twist (Figure 5(a–d)). Taken together, overexpressed miR-509-3p could reverse the promotion of HCC cell malignant biological activities by Twist.

(a) Evaluation of cell proliferation using MTT assay. (b) Determination of cell invasion using Transwell assay. (c) Confirmation of cell migration using wound healing assay. (d) Detection of expression of EMT-related proteins using western blot. **P < 0.01 vs. NC mimics+NC group; ##P < 0.01 vs. miR-509-3p+NC group; &&P < 0.01 vs. NC mimics+Twist group.

### Inhibition of in vivo growth and lung metastasis of hepatocellular carcinoma by miR-509-3p

**Figure 6.**
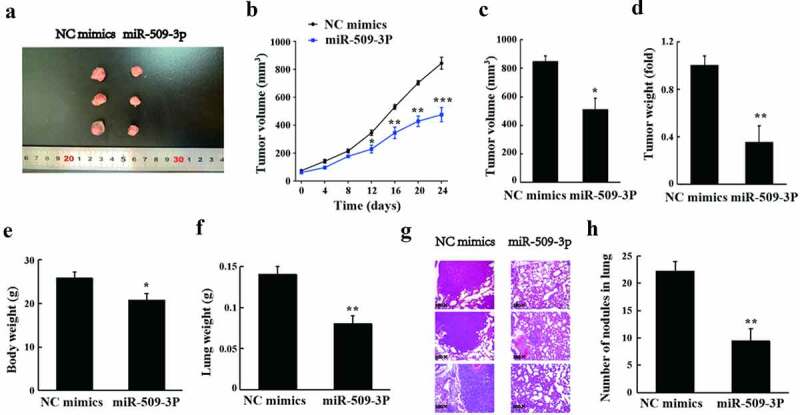
miR-509-3p inhibits the growth and lung metastasis of HCC in vivo

As shown in the results of tumorigenesis in nude mice, tumor volume and weight appeared to reduce after overexpression of miR-509-3p (Figure 6(a–d)). Additionally, a marked overexpressed miR-509-3p-caused reduction also revealed in body weight and lung weight in the mice (Figure 6(e,f)). According to the observation of HE staining, by comparison with the NC mimics group, the metastatic lesions and nodules in the lungs were significantly reduced in the miR-509-3p group (Figure 6(g,h)). Collectively, miR-509-3p could significantly inhibit HCC growth and lung metastasis in vivo.

(a) Ex vivo tumors of nude mice. (b) Changes of tumor volume change of nude mice. (c) Tumor volume of nude mice; (d) Tumor weight of mice in each treatment group. € Body weight of nude mice after lung metastasis test. (f) Lung weight of nude mice after lung metastasis test. (g,h) Observation of pathological changes of lung tissues and tumor nodules in nude mice using HE staining. *P < 0.05, **P < 0.01 and ***P < 0.001 vs. NC mimics group.

## Discussion

Among liver cancer, a common aggressive cancer, HCC contributes the most to the primary liver cancer. The current treatment for HCC, surgical resection, chemotherapy and radiation, cannot avoid postoperative recurrence, tumor metastasis or poor survival due to the invasive and metastatic natures of HCC [22]. Accordingly, for developing novel and effective therapeutic strategies, molecular mechanisms of HCC progression are in urgent need of investigation.

As an essential epigenetic regulator of gene expression in cancer [23], miRNAs can lead to mRNA degradation or translation disruption by binding to the mRNA 3ʹ-UTR, and play complex and diverse functions in cancer development [24]. Taking HCC as an example, in cancer cells, miR-124 targets transcription activator STAT3 to perform induction of apoptosis and inhibition of proliferation, thus suppressing the growth of HCC [25]. MiR-142-3p targets lactate dehydrogenase A to perform inhibition of glycolysis, which thereby results in the suppression of HCC cell proliferation [26]. And miR-122 directly targets the 3‘-UTR binding site of the endogenous apoptosis regulator Bcl-w to cause the reduction of mRNA and protein expression of Bcl-w, which thus inhibiting cancer cell viability and activating caspase-3 and finally promoting HCC cell apoptosis [27]. And research reports confirmed that miR-509-3p was likewise confirmed as an oncogene in many cancers [17]. Nonetheless, its functionality and related mechanisms in HCC remain unclear. Here, through our experiments, a lowly expressed miR-509-3p was found in HCC, and would be further decreased in HCC tissues that developed lymph node metastasis, suggesting the association between miR-509-3p and the development and metastasis of HCC. Additionally, overexpressed miR-509-3p-caused inhibition of the proliferation ability, cell viability, migration ability, invasion and EMT of HCC cells was revealed in in vitro trials, while in vivo experimental results showed the inhibition of the growth and lung metastasis of HCC by overexpression of miR-509-3p.

EMT, a pivotal mechanism for tumor metastasis [28], is manifested as epithelial cell shape changes, acquisition of motility and invasion ability and acquisition of mesenchymal phenotype. Currently, EMT is mainly characterized by down-regulation of E-cadherin and up-regulation of N-cadherin and Vimentin. EMT-caused abnormities of structure and function of adhesion molecules in tumor cells lead to the decrease of adhesion between tumor cells, along with increased motility and a higher probability of invasion or metastasis of tumor cells. The above conclusion is consistent with the findings of this experiment.

Twist, twist family bHLH transcription factor 1, is a critical inducible transcription factor for EMT. Twist regulates the transcription of EMT-related genes through down-regulation of epithelial phenotype-related genes and up-regulation of mesenchymal phenotype-related genes [29]. Through that, Twist participates in promoting cell differentiation, proliferation, metastasis and anti-apoptosis, which is associated with cancer stem cell phenotypes [30,31]. Grzegrzolka et al. pointed out that the activation of EMT by Twist was crucial for breast cancer in situ to develop into a invasive stage [32]. Jing et al. proved that Twist up-regulated Vimentin protein through circRNA-Cul2 and promoted EMT in HCC, indicating that Twist is a critical transcription factor mediating EMT and then promoting tumorigenesis and metastasis [33]. In the results of this trial, Twist was found to be upregulated in HCC, elevating the EMT in HCC. Based on bioinformatics prediction analysis and conformation using dual-luciferase reporter assay, miR-509-3p could target Twist protein, and miR-509-3p expression had a negative relationship with Twist. Further exploration revealed the promotion of HCC development and metastasis by Twist, and this promotion was able to be reversed by miR-509-3p. To conclude, miR-509-3p inhibits metastasis and EMT of HCC by targeting Twist.

## Conclusion

In summary, miR-509-3p was down-regulated in HCC. Overexpressed miR-509-3p can significantly inhibit HCC cell metastasis and EMT by targeting Twist. However, in our study, the exploration of the multifaceted roles of miRNAs, and the relationship between miR-509-3p expression and HCC clinicopathology has limitations. Therefore, comprehensive elucidation of the mechanism of miR-509-3p in HCC metastasis is required further probes.

## Highlights

1. MiR-509-3p is downregulated in hepatocellular carcinoma.
2. MiR-509-3p overexpression inhibit HCC cells proliferation, migration, invasion and epithelial–mesenchymal transition.
3. MiR-509-3p targets Twist to inhibit hepatocellular carcinoma progression.
4. MiR-509-3p inhibit HCC growth and lung metastasis in vivo.
